# Exploring the role of succinyl carnitine in the association between CD39⁺ CD4⁺ T cell and ulcerative colitis: A Mendelian randomization study

**DOI:** 10.1515/med-2025-1240

**Published:** 2025-07-17

**Authors:** Li Chen, Ying Yi, Yun Zhu

**Affiliations:** The Second People’s Hospital of China, Three Gorges University, The Second People’s Hospital of Yichang, Yichang 443000, Hubei, China; The Second People’s Hospital of China, Three Gorges University, The Second People’s Hospital of Yichang, No. 21 Xiling 1st Road, Xiling District, Yichang 443000, Hubei, China

**Keywords:** T cells, succinyl carnitine, UC, Mendelian randomization, preventive treatment

## Abstract

**Objective:**

This study aimed to investigate the potential causal relationship between immune cell and the risk of ulcerative colitis (UC), and to explore whether serum metabolites may mediate this association, thereby suggesting potential biomarkers or therapeutic targets.

**Methods:**

We conducted a Mendelian randomization (MR) analysis using summary statistics from genome-wide association studies to evaluate both the direct effects and potential mediating roles of 731 immune cells and 1,400 serum metabolites in relation to UC. Instrumental variables were rigorously selected based on genome-wide significance and linkage disequilibrium thresholds. The primary analytical method was inverse variance weighted, supplemented by MR-Egger regression and weighted median methods to ensure robustness. Cochran’s *Q* test, MR-Egger intercept, and leave-one-out analysis were employed to evaluate heterogeneity and pleiotropy. Mediation MR analysis was conducted to examine potential metabolite-mediated pathways.

**Results:**

We identified a statistically significant positive causal effect of CD39⁺ CD4⁺ T cell on UC risk (OR = 1.05, 95% CI = 1.03–1.08, *beta_all* = 0.05). Sensitivity analyses confirmed the robustness of this association, and reverse MR analysis indicated no causal effect of UC on CD39⁺ CD4⁺ T cell, suggesting a unidirectional relationship. Mediation analysis further revealed that succinyl carnitine (C4DC) partially mediated the effect of CD39⁺ CD4⁺ T cell on UC, with a mediation proportion of 3.3%.

**Conclusion:**

Our findings suggest that CD39⁺ CD4⁺ T cell may increase the risk of UC, potentially by modulating the levels of succinyl carnitine (C4DC). These results indicate a potential immunometabolic pathway in UC pathogenesis and highlight CD39⁺ CD4⁺ T cell and C4DC as promising targets for further research. However, additional experimental validation is required to confirm these findings and assess their clinical relevance.

## 1 Introduction

Ulcerative colitis (UC) is a chronic, relapsing inflammatory bowel disease characterized by continuous mucosal inflammation of the colon, it typically presents with symptoms such as diarrhea, abdominal pain, and rectal bleeding [1]. Although the precise etiology of UC remains unclear, increasing evidence suggests that its onset and progression result from a complex interplay among genetic susceptibility, environmental triggers, gut microbiota dysbiosis, and immune dysregulation [2]. Due to the absence of standardized and accurate early diagnostic criteria for UC [3], misdiagnosis remains common, which negatively affects patients’ quality of life and leads to substantial medical resource consumption. Identifying novel biomarkers may deepen our understanding of UC pathogenesis and help optimize diagnostic and therapeutic strategies [4].

A central pathological feature of UC is the disruption of intestinal immune homeostasis [5]. Under normal physiological conditions, the gut immune system maintains tolerance to self-antigens and commensal microbiota. In UC, this balance is disturbed, leading to excessive infiltration of immune cells – such as T lymphocytes, B cells, macrophages, and natural killer (NK) cells – into the colonic mucosa [6]. These immune cells secrete pro-inflammatory cytokines (e.g., TNF-α, IFN-γ, IL-17) and chemokines, which contribute to mucosal damage and chronic inflammation [7]. Studies have shown that the restoration of regulatory T cells (Tregs) may support clinical remission in UC patients [8]. For instance, CD4⁺ T cells can differentiate into pro-inflammatory Th1 and Th17 subsets in UC, promoting epithelial injury [9]. Additionally, increased activation of mucosal B cells [10] and macrophages [11] has been associated with disease severity and risk of relapse. Furthermore, genetic factors, including mutations in immune-related genes such as NOD2 and IL-23R, have also been linked to increased susceptibility to UC [12]. Thus, a more comprehensive understanding of immune regulatory mechanisms is essential for elucidating UC pathogenesis and developing novel treatment strategies.

Concurrently, serum metabolites have emerged as important contributors to UC pathogenesis, serving not only as potential biomarkers but also as mediators of disease processes [13,14]. Metabolic dysregulation – especially involving amino acid, lipid, and short-chain fatty acid (SCFA) metabolism – is commonly observed in UC patients [15]. However, observational studies have reported inconsistent findings regarding serum valine and tissue glutamate levels in UC compared to healthy controls [16,17]. Reduced levels of SCFAs are known to impair the integrity of the intestinal epithelial barrier, thereby increasing susceptibility to inflammation [18]. Moreover, certain lipid metabolites, such as lysophosphatidic acid, may exacerbate inflammation by promoting immune cell activation and migration [19]. Despite these findings, the mechanistic links between immune dysregulation and metabolic abnormalities in UC remain poorly understood. While prior studies have independently investigated immune cells and serum metabolites, few have systematically explored their interaction – particularly whether serum metabolites mediate the effect of immune cells on UC risk.

Mendelian randomization (MR), a genetic epidemiological approach, provides an effective tool for causal inference by using genetic variants as instrumental variables (IVs). It helps to minimize confounding and reverse causality that often limit traditional observational studies [20]. In this study, we employ a comprehensive two-sample MR framework to explore the causal relationships among immune cells, serum metabolites, and UC risk. Specifically, we aim to identify immune cells that contribute to UC pathogenesis through metabolite-mediated pathways. By elucidating these immunometabolic interactions, our findings may offer novel mechanistic insights and suggest potential targets for the prevention and treatment of UC.

## 2 Materials and methods

### 2.1 Study design

**Figure 1:**
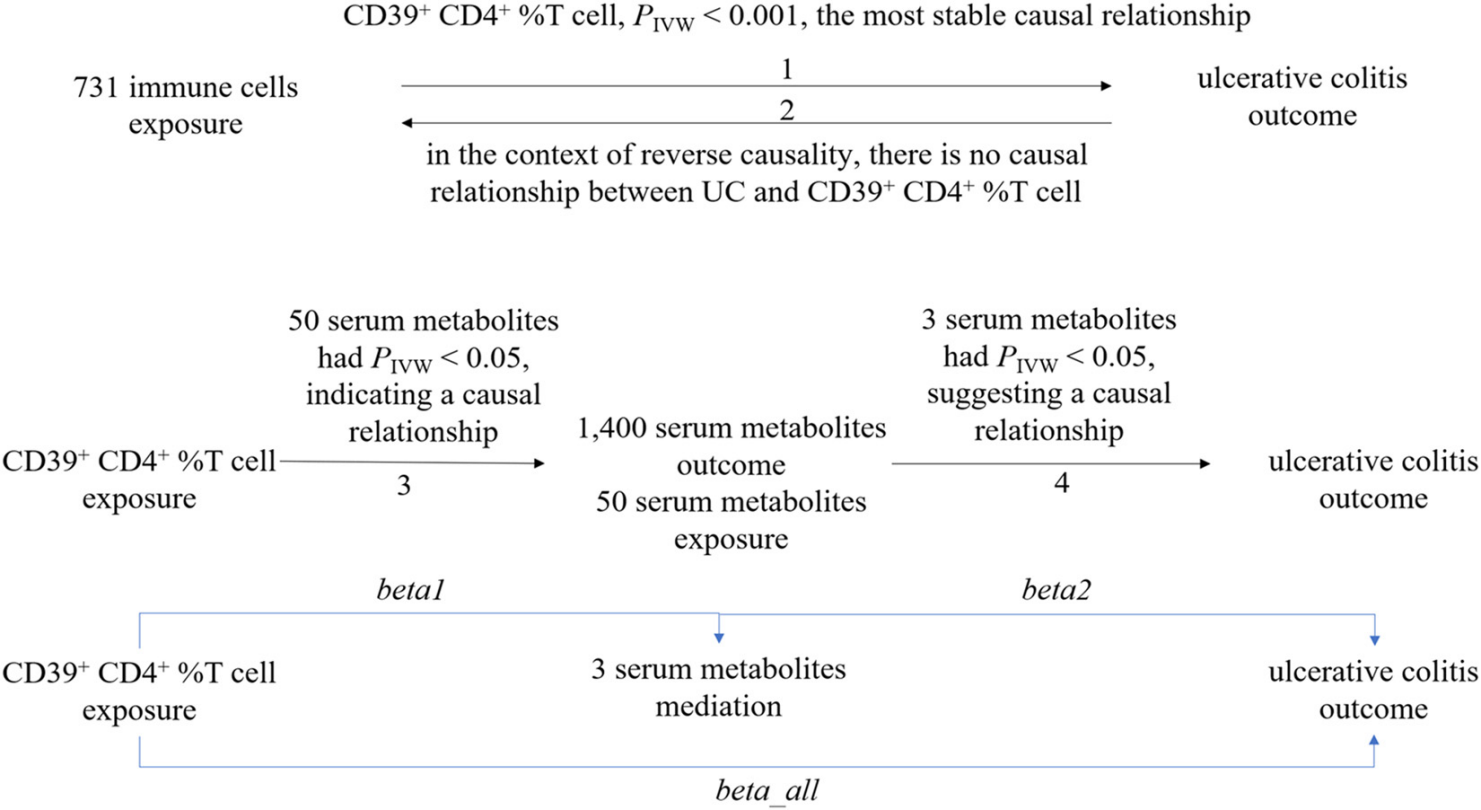
MR study design investigating the effects of 731 immune cells on UC mediated by 1,400 serum metabolites.

This study employed a two-sample MR design to investigate the causal relationship between 731 immune cells as exposure factors and UC as the outcome, as well as the potential mediating role of 1,400 serum metabolites. The specific analytical steps are summarized in Figure 1. (1) Analyze the unidirectional causal relationship between 731 immune cells as exposure factors and UC as the outcome variable, and identify the most robust immune cell associated with UC (CD39⁺ CD4⁺ %T cell. In this study, the trait “CD39⁺ CD4⁺ %T cell” refers to the proportion of CD4⁺ T cells that express CD39^+^, as defined in the original genome-wide association studies (GWAS) dataset. For clarity, we refer to this trait as “CD39⁺ CD4⁺ T cells” throughout the manuscript when discussing biological interpretation). (2) Conduct reverse MR analysis using UC as the exposure and the identified immune cell as the outcome to confirm the absence of reverse causality. (3) Analyze the causal relationship between the selected immune cells as exposure factors and 1,400 serum metabolites as outcome variables. (4) Analyze the causal relationship between 50 serum metabolites that have a causal relationship with the selected immune cells and UC as the outcome variable. Finally, calculate the total effect (*beta_all*) of the selected immune cells (CD39⁺ CD4⁺ T cell) on UC, the effect of the selected immune cells on the identified mediating serum metabolites (*beta*1), and the effect of the mediating serum metabolites on UC (*beta*2), in order to determine the mediating effect and mediation effect ratio of the serum metabolites in the influence of the selected immune cells on UC.

### 2.2 Data sources

#### 2.2.1 Immune cell data

Summary statistics for 731 immune cells were obtained from the GWAS catalog (Registration Numbers GCST90001391–GCST90002121) [21]. The study data were derived from 3,757 European individuals, after adjusting for confounders such as age and sex, more than 22 million single nucleotide polymorphisms (SNPs) were genotyped using high-density arrays.

#### 2.2.2 Serum metabolite data

A total of 1,400 serum metabolites were sourced from a Canadian aging longitudinal cohort study involving 8,299 participants [22], which included 1,091 metabolites and 309 metabolite ratios. These metabolites were categorized into eight major classes: amino acids, carbohydrates, cofactors and vitamins, energy metabolites, lipids, nucleotides, peptides, and xenobiotic metabolites. Detailed GWAS data are available through the GWAS Catalog (Registration Numbers: GCST90199621–GCST90204063).

#### 2.2.3 UC data

UC summary statistics were obtained from the FinnGen project (R11 release), a nationwide public–private collaboration in Finland aimed at exploring the etiology of various diseases [23]. This dataset includes information from 6,435 UC patients and 446,419 control individuals. All data used in this study were derived from publicly available databases and did not involve direct patient contact or ethical approval.

### 2.3 Selection of IVs

SNPs were selected as IVs according to the three core assumptions of MR [24]: (1) SNPs significantly associated with the exposure factor are selected as IVs. (2) SNPs should be independent of known confounders, ensuring relative independence. (3) SNPs should influence the outcome variable solely through the exposure factor. Following previous similar studies [25–27], in order to ensure the integrity and accuracy of causal inference, this study applied a threshold of *P* < 1 × 10⁻⁵ when screening for SNPs associated with the 731 immune cells and 1,400 serum metabolites [28]. In order to remove SNPs in linkage disequilibrium and avoid statistical biases caused by genetic linkage, the study set parameters of *R*² = 0.001 and a 10,000 kb window [29]. Palindromic SNPs with intermediate allele frequencies were excluded to ensure strand alignment [30]. Finally, the strength of each instrument was evaluated using the *F*-statistic, with SNPs having *F* > 10 retained to minimize weak instrument bias [31].

### 2.4 Statistical methods

All statistical analyses were performed using R software (version 4.3.2), with the “Two-SampleMR” and “MR-PRESSO” packages. The inverse variance weighted (IVW) method was employed as the primary analytical approach, as it is widely accepted for causal effect estimation [32]. *P* < 0.05 was considered statistically significant [33], MR-Egger regression and the weighted median method were conducted as complementary analyses to assess robustness [34]. Heterogeneity was assessed using Cochran’s *Q* test under both the IVW and MR-Egger methods, with *P* > 0.05 indicating no significant heterogeneity. Horizontal pleiotropy was evaluated using the MR-PRESSO global test and the MR-Egger intercept test, where *P* > 0.05 was interpreted as absence of pleiotropy [35]. A leave-one-out sensitivity analysis was also performed to evaluate the influence of individual SNPs on the overall causal estimate. The effect relationships between exposures and outcomes were evaluated using odds ratios (OR) and their 95% confidence intervals (CI).

A two-step mediation MR analysis was conducted to estimate the total effect (*beta_all*) of selected immune cells on UC, the effect of immune cells on serum metabolites (*beta*1), and the effect of serum metabolites on UC (*beta*2). From these, the indirect mediation effect (*beta*12), direct effect (*beta_dir*), and mediation proportion (*beta*12*_p*) were calculated [36]. Specifically, the total effect represents the overall impact of selected immune cells on UC, which can be decomposed into the direct effect of selected immune cells on UC and the indirect effect mediated by the identified serum metabolites. The mediation effect (*beta*12 *= beta*1 *× beta*2) represents the indirect effect of selected immune cells on UC through the serum metabolites. The direct effect (*beta_dir = beta_all − beta*12) represents the direct impact of selected immune cells on UC. The mediation effect ratio (*beta*12*_p = beta*12*/beta_all*) reflects the proportion of the total effect that is mediated by the serum metabolites, providing insight into the percentage of UC risk influenced by selected immune cells through these metabolites. If the directionality of *beta*12 and *beta_all* is inconsistent (i.e., OR > 1 or < 1), the mediation proportion is not interpretable, and a one-sided test with a significance level of *α* = 0.05 was applied.

## 3 Results

### 3.1 Causal relationship between 731 immune cells and UC

After excluding immune cells with linkage disequilibrium and weak IVs, two-sample MR analysis was performed using immune cells as exposures and UC as the outcome. The IVW method identified 52 immune cells with *P*-values < 0.05. Further evaluation of heterogeneity and horizontal pleiotropy revealed that 27 of these immune cells exhibited significant heterogeneity (*P* < 0.05), including the following: myeloid DC %DC, DC AC, CD62L^−^ DC %DC, CD86^+^ myeloid DC %DC, CD62L^−^ myeloid DC AC, CD62L^−^ myeloid DC %DC, CD62L^−^ CD86^+^ myeloid DC %DC, CD33dim HLA DR^+^ CD11b^+^ AC, CD14^+^ CD16^+^ monocyte AC, T/B, NK %CD3^−^ lymphocyte, HLA DR^+^ NK AC, HLA DR^+^ NK %NK, HLA DR^+^ NK %CD3^−^ lymphocyte, CD127^−^ CD8br %T cell, CD45RA^−^ CD28^−^ CD8br %T cell, CD19 on sw mem, CD28 on CD45RA^−^CD4 not Treg, CD86 on myeloid DC, CD16 on CD14^−^ CD16^+^ monocyte, HLA DR on CD14^−^ CD16^+^ monocyte, CX3CR1 on CD14^+^ CD16^−^ monocyte, PDL-1 on monocyte, HLA DR on plasmacytoid DC, HLA DR on DC, and HLA DR on CD33dim HLA DR^+^ CD11b^−^, HLA DR on CD33^−^ HLA DR^+^.

**Figure 2:**
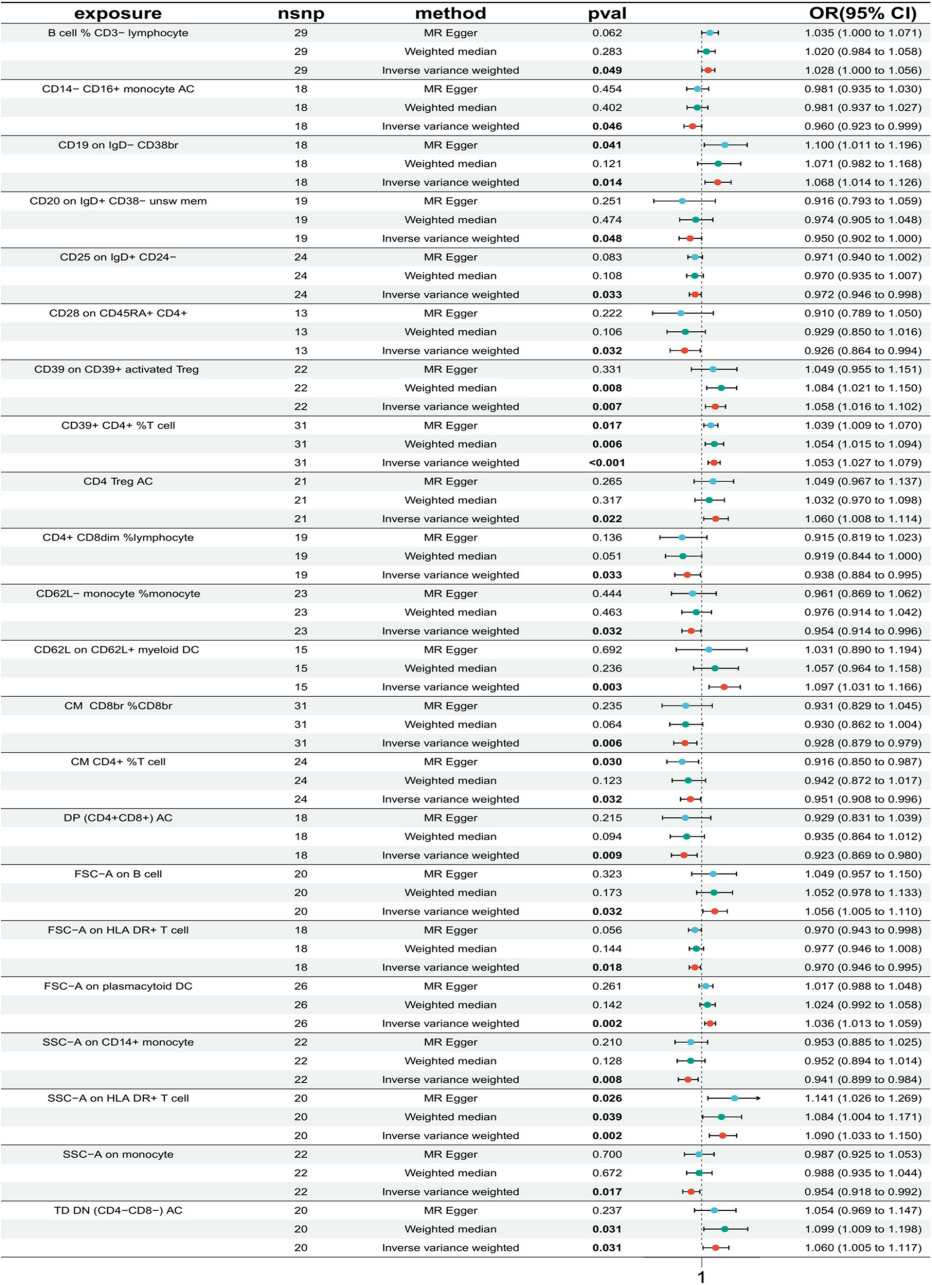
Forest plot of MR analysis between immune cells and UC. The black line represents the 95% CI of the effect size; the red, green, and blue dots represent effect estimates obtained from different MR methods; exposure: immune cell phenotypes; nsnp: number of SNPs; method: analytical method used; *P*_val_: *P*-value.

Additionally, two immune cells (IgD^+^ CD38^−^ %B cell, EM CD8br %T cell) showed evidence of horizontal pleiotropy (*P* < 0.05). Another immune cell (CD45RA^−^ CD28^−^ CD8br AC) had an OR close to 1 and was thus excluded. After excluding these 30 immune cells mentioned above, a forest plot of the remaining 22 immune cells was generated using three MR methods (Figure 2).

To further assess the reliability of the results, this study combined MR-Egger and weighted median analyses. It was found that two immune cells exhibited a more reliable causal relationship with UC. Specifically, immune cells (CD39^+^ CD4^+^ %T cell, SSC-A on HLA DR^+^ T cell) demonstrated *P*_IVW_ < 0.05, with both MR-Egger and weighted median methods showing *P* < 0.05. After comparing the *P*_IVW_ values, the study ultimately selected the immune cell with the most stable causal relationship (CD39^+^ CD4^+^ %T cell, *P* < 0.001) for subsequent analysis.

### 3.2 Reverse MR

To meet the requirements for the mediation MR study, this research conducted a reverse MR analysis with UC as the exposure and immune cells (CD39^+^ CD4^+^ %T cell) as the outcome. The results showed that all five analytical methods had *P* ＞0.05, indicating no statistical significance. This suggests that, in the context of reverse causality, there is no causal relationship between UC and CD39^+^ CD4^+^ %T cell (Table 1).

**Table 1:** Reverse MR analysis of UC and CD39^+^ CD4^+^ %T cell

Method	nsnp	*β*	se	*P*_val_	or	or_lci95	or_uci95
MR Egger	40	0.05	0.10	0.60	1.05	0.87	1.28
Weighted median	40	0.05	0.04	0.37	1.04	0.95	1.14
IVW	40	0.03	0.03	0.37	1.03	0.97	1.10
Simple mode	40	0.08	0.08	0.44	1.06	0.91	1.24
Weighted mode	40	0.05	0.07	0.42	1.06	0.93	1.21

β = beta, se = standard error.

### 3.3 Causal relationship between CD39^+^ CD4^+^ %T cell and 1,400 serum metabolites

To investigate the causal relationship between the most stable immune cell (CD39^+^ CD4^+^ %T cell) and serum metabolites in UC, the study conducted 1,400 two-sample MR analyses after removing linkage disequilibrium and weak IVs. In the analysis, CD39^+^ CD4^+^ %T cell was used as the exposure and 1,400 serum metabolites as the outcomes.

**Figure 3:**
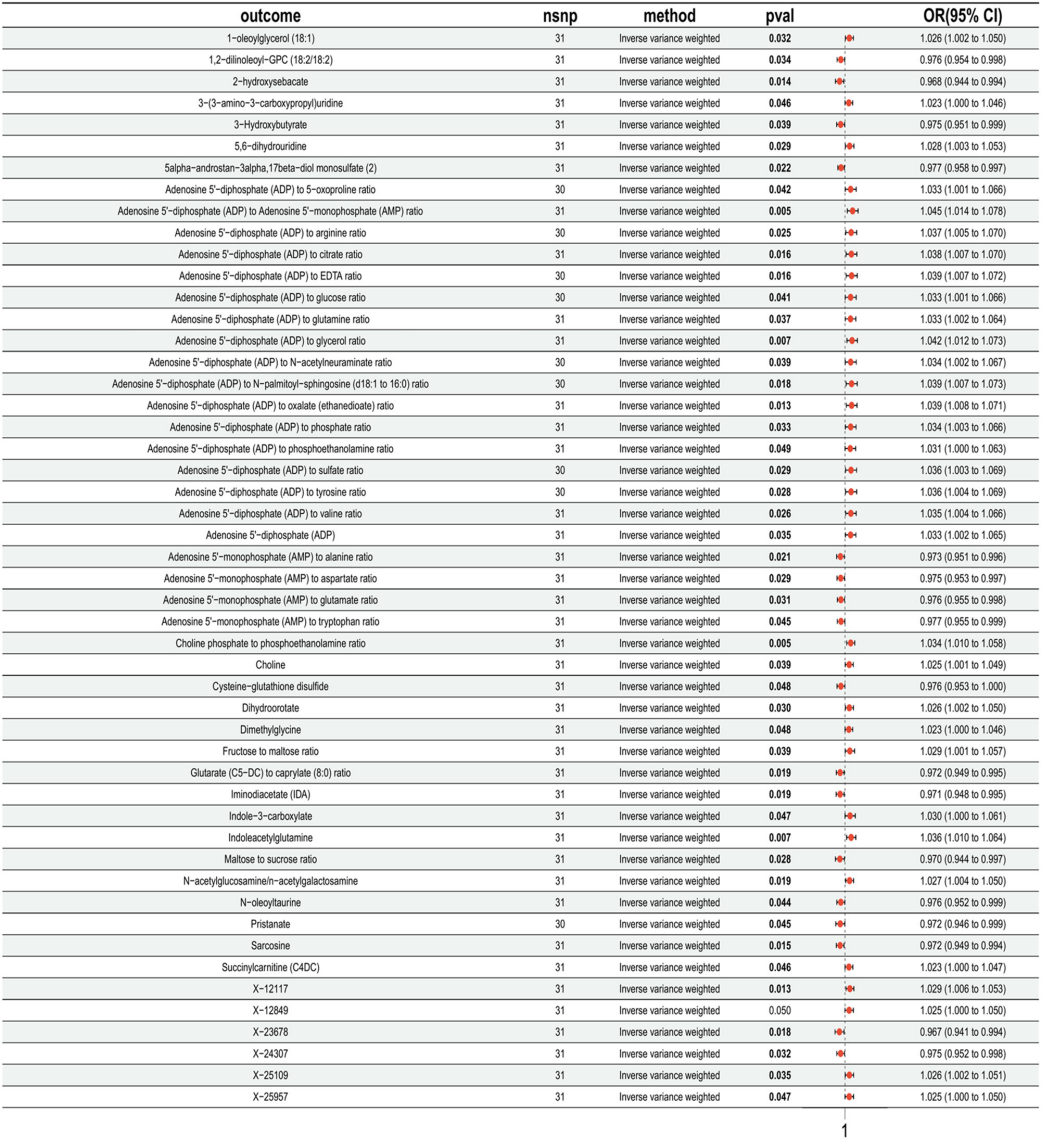
Forest plot of MR analysis between CD39^+^ CD4^+^ %T cell and serum metabolites. The black line represents the 95% CI of the effect size; the red dot represents the effect size; exposure: immune cell phenotypes; nsnp: number of SNPs; method: analytical method used; *P*_val_: *P*-value.

The results revealed that 51 serum metabolites had *P*_IVW_ < 0.05, indicating a significant causal relationship. To further validate the reliability of the results, heterogeneity and horizontal pleiotropy tests were performed. The analysis found that the serum metabolite (13^−^HODE + 9^−^HODE) displayed heterogeneity (*P* < 0.05). After excluding this serum metabolite, a forest plot of 50 serum metabolites from the IVW analysis method was generated (Figure 3).

### 3.4 Causal relationship between 50 serum metabolites associated with CD39^+^ CD4^+^ %T cell and UC

After removing serum metabolite linkage disequilibrium and weak IVs, this study conducted 50 two-sample MR analyses with 50 serum metabolites as the exposures and UC as the outcome. The results revealed that three serum metabolites had *P*_IVW_ < 0.05, suggesting a causal relationship with UC. Sensitivity analysis further confirmed the reliability of the results, and no evidence of heterogeneity or horizontal pleiotropy was observed. Specifically: 1-oleoylglycerol (18:1) showed a negative correlation with UC (OR = 0.92, 95% CI = 0.85–1.00). Succinyl carnitine (C4DC) showed a positive correlation with UC (OR = 1.08, 95% CI = 1.01–1.15). 1,2-Dilinoleoyl-GPC (18:2/18:2) showed a positive correlation with UC (OR = 1.25, 95% CI = 1.12–1.38). The remaining 47 serum metabolites showed no significant association with UC (*P*_IVW_ > 0.05).

### 3.5 Mediation of the effect of immune cells on UC by serum metabolites

A two-step mediation MR analysis was conducted to explore whether serum metabolites mediated the effect of CD39⁺ CD4⁺ T cell on UC: (1) The analysis confirmed a positive correlation between CD39^+^ CD4^+^ %T cell and UC (OR = 1.05, 95% CI = 1.03–1.08, *β_all* = 0.05). Additionally, CD39^+^ CD4^+^ %T cell were positively correlated with 1-oleoylglycerol (18:1) (OR = 1.03, 95% CI = 1.00–1.05, *β*1 = 0.03). However, 1-oleoylglycerol (18:1) was negatively correlated with UC (OR = 0.92, 95% CI = 0.85–1.00, *β*2 = −0.08). The total effect of exposure on the outcome (*β_all* = 0.05) and the mediation effect (*β*12 = −0.002) were inconsistent in direction, suggesting that exposure might not influence the outcome through this mediator. (2) The analysis found a positive correlation between CD39^+^ CD4^+^ %T cell and UC (OR = 1.05, 95% CI = 1.03–1.08, *β_all* = 0.05). CD39^+^ CD4^+^ %T cell were positively correlated with succinyl carnitine (C4DC) (OR = 1.02, 95% CI = 1.00–1.05, *β*1 = 0.02). Succinyl carnitine (C4DC) was also positively correlated with UC (OR = 1.08, 95% CI = 1.01–1.15, *β*2 = 0.08). The total effect of exposure on the outcome (*β_all* = 0.05) and the mediation effect (*β*12 = 0.002) were consistent in direction, with the mediation effect ratio (β12_p) being 3.3%. This suggests that succinyl carnitine (C4DC) mediates the relationship between CD39^+^ CD4^+^ %T cell and UC, contributing to the increased risk of UC. (3) CD39^+^ CD4^+^ %T cell were positively correlated with UC (OR = 1.05, 95% CI = 1.03–1.08, *β_all* = 0.05). 1,2-Dilinoleoyl-GPC (18:2/18:2) was negatively correlated with CD39^+^ CD4^+^ %T cells (OR = 0.98, 95% CI = 0.95–1.00, *β*1 = −0.02). However, 1,2-dilinoleoyl-GPC (18:2/18:2) was positively correlated with UC (OR = 1.25, 95% CI = 1.12–1.38). The total effect of exposure on the outcome (*β_all* = 0.05) and the mediation effect (*β*12 = −0.005) were inconsistent in direction, indicating that exposure might not influence UC through this mediator.

## 4 Discussion

CD39 (ectonucleoside triphosphate diphosphohydrolase1, ENTPD1) is widely expressed on T cells, regulatory T cells (Tregs), and certain innate immune cells [37]. Its primary function is to hydrolyze adenosine triphosphate and adenosine diphosphate into adenosine monophosphate, which is subsequently converted into immunosuppressive adenosine by CD73. Adenosine, in turn, binds to adenosine receptors – such as the A2A receptor – thereby inhibiting immune cell activation and reducing the release of pro-inflammatory cytokines, including IL-1β and TNF-α. This mechanism plays an important role in modulating inflammatory responses. Studies [38] have shown that CD39 expression is upregulated in certain inflammatory diseases, and dysregulation of its immunosuppressive function may contribute to aberrant immune responses and the persistence of chronic inflammation.

CD4^+^ T cells [39], as helper T (Th) cells, play a pivotal role in adaptive immune responses by secreting cytokines and activating other immune cells. Previous studies have demonstrated that activated memory CD4⁺ T cells and follicular helper T cells are particularly involved in promoting immune activation and inflammation in UC [40]. In UC patients, CD39⁺ CD4⁺ T cell may disrupt intestinal immune homeostasis by altering the balance between Tregs and effector T cells. This imbalance can result in excessive immunosuppression, impaired immune surveillance of gut microbiota, and exacerbated intestinal inflammation. Moreover, these cells may aggravate disease progression by releasing pro-inflammatory cytokines or through direct interaction with the intestinal microenvironment [41].

Our mediation MR analysis further suggests that CD39⁺ CD4⁺ T cell may influence UC risk by regulating the level of succinyl carnitine (C4DC). Succinyl carnitine [42] is a carnitine ester formed through conjugation with succinyl-CoA, a key intermediate in the tricarboxylic acid (TCA) cycle. Carnitine derivatives [43], including C4DC, facilitate the mitochondrial transport of fatty acids and their intermediates, bridging β-oxidation and the TCA cycle. C4DC levels are closely linked to T-cell metabolic adaptation, energy metabolism, oxidative stress, and immune regulation. CD39⁺ CD4⁺ T cell may affect mitochondrial metabolism by influencing the conversion of succinyl-CoA into C4DC. Accumulation of C4DC may contribute to UC-associated inflammation through the following mechanisms: (1) overactivation of effector T cells, (2) increased release of pro-inflammatory cytokines (e.g., IL-17, IFN-γ), and (3) enhanced oxidative stress leading to disruption of the intestinal epithelial barrier [44]. Moreover, altered C4DC levels may modulate immune signaling pathways, affecting T-cell functionality and the stability of the intestinal microenvironment. In particular, CD39⁺ CD4⁺ T cell – which represent a subpopulation of CD4⁺ T cell expressing the immunosuppressive ectoenzyme CD39 – may exacerbate immune dysregulation and chronic inflammation in the gut, thereby promoting UC pathogenesis and progression.

Despite providing novel insights into the immunometabolic mechanisms of UC, this study has several limitations. (1) Although the MR approach effectively reduces confounding and reverse causation, the validity of its conclusions relies on the strength and assumptions of the IVs. (2) The prevalence and clinical features of UC vary significantly across ethnic groups [45]. For example, UC is more prevalent in Western than in Asian populations, and within Western cohorts, it is more common among Caucasians than Black individuals. These differences suggest that the generalizability of our findings may be limited, and replication in diverse populations is warranted. (3) While C4DC was identified as a key metabolite-linking metabolism and immune function, its specific biological mechanisms remain incompletely understood. Most existing studies focus on indirect associations; further experimental validation is needed to clarify its direct role. (4) The mediation effect ratio observed in this study was only 3.3%, indicating that C4DC may be just one of multiple intermediates involved. Other potential mediators and pathways should be explored in future research. (5) The immune cell data used in this study were derived from peripheral blood. However, immune cell phenotypes and functions in the gastrointestinal tract may differ. Thus, peripheral measurements may not fully reflect gut-specific immune responses. Future studies should consider directly analyzing mucosal immune cells for more tissue-relevant insights.

To build upon these findings, future research should integrate clinical and experimental approaches to investigate the biological mechanisms through which CD39⁺ CD4⁺ T cell regulate UC via C4DC. Large-scale cohort studies and multi-center collaborations will be essential for validating our results and assessing their clinical relevance. This study provides a theoretical foundation for understanding the immune-metabolic network in UC and may offer novel targets and strategies for disease prevention and therapeutic intervention.

## 5 Conclusion

This study is the first to apply a mediation MR approach to investigate the causal relationships among immune cells, serum metabolites, and UC. Our findings reveal a positive causal association between CD39⁺ CD4⁺ T cell and UC, which was supported by multiple sensitivity analyses. Reverse MR analysis did not identify a causal effect of UC on CD39⁺ CD4⁺ T cell, suggesting a unidirectional influence of these cells on disease risk. Moreover, the mediation MR analysis indicated that CD39⁺ CD4⁺ T cell may contribute to UC pathogenesis by modulating the levels of succinyl carnitine (C4DC), with a mediation effect proportion of 3.3%.

These findings provide novel insights into the immunometabolic mechanisms underlying UC and offer a new perspective for future mechanistic studies and therapeutic target exploration in this field.

## Abbreviations

CI: confidence intervals
GWAS: genome-wide association studies
IVs: instrumental variables
IVW: inverse variance weighted
MR: Mendelian randomization
NK: natural killer
OR: odds ratios
SCFA: short-chain fatty acid
SNPs: single nucleotide polymorphisms
UC: ulcerative colitis

## Supplementary Material

Supplementary Table 1

Supplementary Table 2
